# Supplementary material for: Multi-site comparison of factors influencing progress of African insecticide testing facilities towards an international Quality Management System certification
Source: PLoS One. 2021 Nov 15;16(11):e0259849. doi: 10.1371/journal.pone.0259849 (PMC8592480; doi:10.1371/journal.pone.0259849)
Supplement: S1 File — (DOCX) [file pone.0259849.s001.docx]

**Sustainable capacity in Africa for vector control research and product development**

Interview toolkit^[[1]](#footnote-1)^

This is the full toolkit of questions; however, individuals will only be asked a subset of questions dependent on their role and experience.

**Question Guide**

Question 1: What is your role at [INSTITUTION]?

- How were you recruited for/identified for this role?
- What training did you receive?
- What do you see as your future in this rile?
- What motivates you in this role?
- Who do you need to support you to do this role well?
- Which (if any) computer systems do you use in your role at [INSTITUTION]?
- Can you describe how you use computer systems in your role?

Question 2: Can you tell me about your role in obtaining GLP certification at [INSTITUTION]?

- What is your involvement with GLP studies?
- What was your involvement with the GLP certification process?
- Which activities related to GLP were most critical for success?
  - Why was this?
- What training did you receive related to GLP?
  - Was it helpful?
  - Was anything missing?
  - Was it timely?
- How was training related to GLP organised at [INSTITUTION]?
- How did you plan the GLP project, how did you determine timelines?

Question 3: How have you personally found the process of working towards GLP certification?

- What were the simplest things to implement related to GLP?
- What were the most successful activities you implemented related to GLP?
- Which roles are most critical for successfully achieving GLP certification?
  - Why?
  - When should they be recruited?
  - How would you recommend they are recruited?
  - Which were the most challenging roles to fill in relation to GLP?
- What barriers were there to GLP certification and how were they overcome?
- What were the main causes of delay in your progress on GLP activities?
- Were there any activities that needed to be completed by other groups or individuals before you could begin progress on GLP activities?
  - What were they?
  - Who was responsible for them?
  - Why?
- Where there any barriers in the systems at [INSTITUTION]?
  - Were these systems changed as part of GLP process?
  - If so how?

**Role specific probes, to be selected depending on role outlined in Question 1/2:**

*SOP Probes*

- *What input did you have into SOP development at [INSTITUTION]?*
  - *Who was involved?*
  - *What was the process?*
  - *Which SOPs were developed first? Why?*
  - *Which SOPs were developed last? Why?*

*Quality Assurance Probes*

- *What role does quality assurance have in your job?*
  - *What do you think of it in relation to your job?*

*Computer Systems Probes*

- *What e-systems are currently in place at [INSTITUTION]?*
- *What e-systems or hardware are needed?*
  - *How could they be sourced?*
- *How are e-systems maintained and managed?*

*Procurement Probes*

- *What was the process for procuring materials and resources needed for GLP certification?*
- *How efficient was this process?*
- *What, if anything, slowed down this process?*
- *What opportunities are there for speeding up this process?*

*Lab Probes*

- *What activities was it necessary to undertake before you could progress to working towards GLP-certification?*
- *Were there any GLP activities you could make progress on independent of wider laboratory improvements of changes?*

Question 4: In your mind, what is the purpose of GLP?

- Why did [INSTITUTION] seek GLP certification?
- What effect does GLP have on your work?
- What effect does GLP have on [INSTITUTION]’s operations?

Question 5: If you were to undertake this GLP certification project again what would you do differently?

- What were the most challenging aspects of the GLP certification process?
- Were there any activities that were related to GLP certification that you felt were unnecessary?
- What could the wider team have done differently?
- What resources or support would have made the process easier?

Question 6: What will GLP certification do for [INSTITUTION] in the future? (MANAGEMENT ONLY)

- What control does the laboratory have over its finances in relation to the wider institution?
- What financial data to you have access to? How is it used (in decision making)?
- How does GLP fit into the labs overall strategy?
- Are strategic plans costed?
- Has maintaining GLP been costed?
- What additional work has been/will be brought in because of GLP?
- What (potential) partnerships will/have be(en) formed as a result of GLP
- What is the financial risk of GLP to the lab?
- What are the indirect benefits of GLP to the organisation (equipment, system improvements etc)?
- (How) do you use GLP in your lab

Question 7: What key lessons have you learnt from the process of working towards GLP certification?

- What advice would you give to other institutions going for GLP certification?

1. Version 3 16/05/18 [↑](#footnote-ref-1)
